# Coxiella burnetii-Associated Endocarditis in a Saudi Adolescent: A Rare Encounter

**DOI:** 10.7759/cureus.50470

**Published:** 2023-12-13

**Authors:** Raghad Hijazi, Mohammed Aldahmash, Abdulmajeed Almansouf, Raghad Alhuthil, Suliman Aljumaah

**Affiliations:** 1 College of Medicine, Alfaisal University, Riyadh, SAU; 2 College of Medicine, King Saud Bin Abdulaziz University for Health Sciences, Riyadh, SAU; 3 Pediatrics, King Faisal Specialist Hospital and Research Centre, Riyadh, SAU; 4 Pediatric Infectious Diseases, King Faisal Specialist Hospital and Research Centre, Riyadh, SAU

**Keywords:** hydroxychloroquine, doxycycline, endocarditis, q fever, coxiella burnetii

## Abstract

Here, we present the case of a 15-year-old Saudi male with a history of cardiac interventions who initially presented with persistent fever. The patient’s complex medical history, including cardiac procedures and recent antibiotic use, added layers to the diagnostic challenge. Despite initial empirical antibiotic therapy, persistent fever prompted further investigations, leading to the identification of vegetation causing right ventricular outflow tract obstruction. *Coxiella burnetii* serology confirmed Q fever infective endocarditis. Tailored antimicrobial therapy, including doxycycline, ciprofloxacin, and hydroxychloroquine, resulted in clinical improvement. During the last visit, he remained well, with a painless rash resolved. His parents were satisfied with the progress. Ongoing antimicrobial therapy, periodic ophthalmology assessments, and potential surgical interventions were planned.

## Introduction

Q fever is a zoonotic disease caused by the obligate intracellular bacterium *Coxiella burnetii*, primarily affecting domesticated animals such as goats, sheep, and cattle [1]. This bacterium can be shed in amniotic fluid, placenta, urine, feces, and milk of the infected animals, leading to environmental contamination [2]. Humans typically get infected with *C. burnetii* by inhaling dust or aerosols that have been contaminated with this bacterium, and, rarely, the infection can occur by consuming unpasteurized milk, tick bites, and person-to-person transmission [3].

Q fever primarily manifests in acute and chronic forms. Typically, patients with acute Q fever present with flu-like symptoms, malaise, headache, atypical pneumonia, and hepatitis lasting one to three weeks with an incubation period of 14 to 39 days [4,5]. In approximately 1-5% of the patients, Q fever becomes localized and chronic [6]. In chronic Q fever, the most common manifestation is infective endocarditis, followed by vascular prosthesis and existing aneurysm infections [7].

Moreover, as children rarely come into contact with livestock, they are typically not in danger. National surveillance records about Q fever from several nations indicate that children and young adults account for a very small proportion of recorded cases. Approximately 3.4% of cases of Q fever in the United States recorded between 2000 and 2012 involved people 18 years of age or younger [8]. However, determining the actual frequency of this disease poses a challenge, often being underestimated due to its clinical variability.

Here, we present the case of a 15-year-old Saudi male with a history of cardiac interventions, who initially presented with persistent fever and was ultimately diagnosed with Q fever infective endocarditis.

## Case presentation

A 15-year-old Saudi male accompanied by his father presented to the King Faisal Specialist Hospital Emergency Department on December 30, 2022, complaining of a persistent, high-grade fever (tympanic 40°C) during the preceding three weeks, characterized by intermittent episodes. The patient had a known medical history of pulmonary atresia, ventricular septal defect, and major aortopulmonary collaterals. Notably, he had undergone augmentation of the pulmonary arteries, unifocalization of major aortopulmonary collaterals, and repair of a right coronary artery fistula on February 28, 2009. The patient had undergone a successful pulmonary valve implantation in 2019 using a 22 mm Melody transcatheter pulmonary valve to treat pulmonary regurgitation. Post-intervention angiography of the prosthetic valve showed the valve in a good position with no impingement on the adjacent structures.

His symptoms included cough and right-sided chest pain lasting for a few seconds, with no reported history of palpitations, diaphoresis, syncope, or dizziness. In addition, the patient had an upper respiratory tract infection, for which he received a five-day course of cefixime on December 10, 2022, at a local hospital in Riyadh. A few days after starting the antibiotics, he developed a rash on his inner thighs. There was no history of exposure to farm animals or sick contacts. There was a history of travel to a sheep grazing area a few days before the acquisition of the infection. During the physical examination, the patient appeared comfortable with no signs of distress. However, an erythematous papular rash was observed bilaterally on the inner thighs. Cardiovascular examination revealed audible S1 and S2 heart sounds with a notable holosystolic murmur heard at the fourth left intercostal space. Chest examination indicated equal bilateral air entry with normal vesicular breathing. Furthermore, increased ear wax bilaterally with mild bulging of the tympanic membrane was noted, along with mild throat erythema. There was no tonsillar enlargement during the throat examination. Poor dental hygiene was also observed.

**Figure 1:**
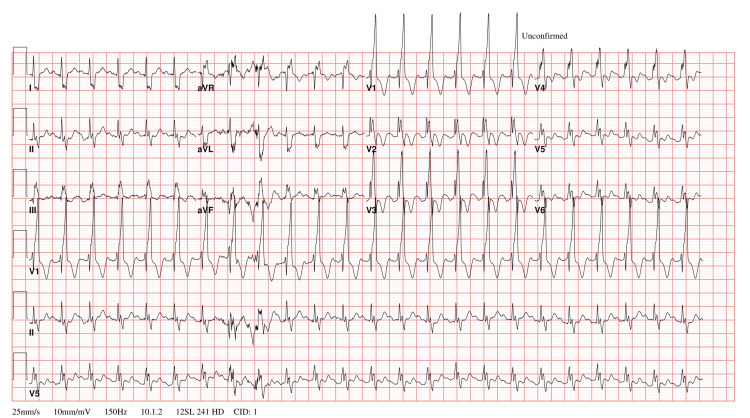
The electrocardiogram showing sinus tachycardia and right bundle branch block. Characteristic rsr' complex in leads V1 and V2.

Three sets of blood cultures were obtained from the patient, and subsequent investigations (see Table 1) revealed abnormalities in his complete blood count and differential (CBCD), showing a normal white blood cell count, low hemoglobin, and hypochromic microcytic anemia. Thrombocytopenia was present. Inflammatory markers showed a normal procalcitonin level and a high C-reactive protein (CRP) level. A respiratory polymerase chain reaction (PCR) test was positive for rhinovirus/enterovirus, while blood, urine, and throat cultures were negative. His chest X-ray did not reveal significant abnormalities. Meanwhile, the electrocardiogram showed sinus tachycardia and right bundle branch block, a characteristic rsr' complex in leads V1 and V2 (see Figure 1). A skin biopsy was also taken from the skin lesions, which came back negative for vasculitis.

**Table 1:** Laboratory results. ND: not done

Lab investigation	At admission (30/12/2022)	One week after the initiation of vancomycin and ceftriaxone (5/1/2023)	One week after the initiation of doxycycline and hydroxychloroquine (22/1/2023)	At discharge (26/1/2023)	One month after the initiation of ciprofloxacin (15/5/2023)	Reference range
White blood cell count	4.1 × 10^9^/L	5.21 × 10^9^/L	3.27 × 10^9^/L	3.95 × 10^9^/L	4.63 × 10^9^/L	3.9–11 × 10^9^/L
Hemoglobin	90 g/L	104 g/L	91 g/L	111 g/L	111 g/L	135–180 g/L
Mean corpuscular volume	74.8 fL	72.5 fL	75.1 fL	74.4 fL	76.4 fL	75–95 fL
Platelet count	139 × 10^9^/L	144 × 10^9^/L	108 × 10^9^/L	103 × 10^9^/L	126 × 10^9^/L	155–435 × 10^9^/L
Procalcitonin	0.12 ng/mL	0.14 ng/mL	ND	0.08 ng/mL	ND	<0.35 ng/mL
C-reactive protein	34 mg/L	36.5 mg/L	9 mg/L	11.1 mg/L	7.2 mg/L	1–3 mg/L
IgG phase two antibodies	>500 U/mL	ND	ND	ND	ND	<20 U/mL

**Figure 2:**
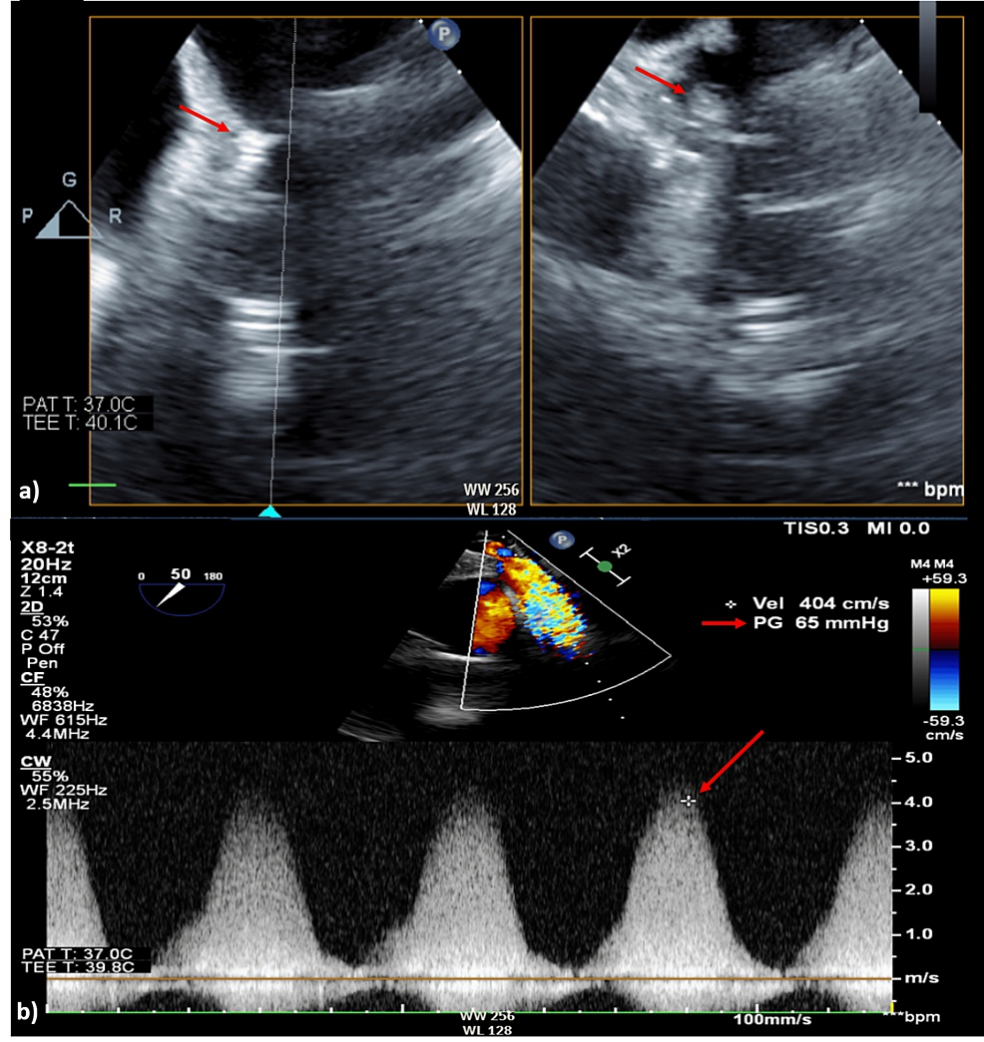
Transthoracic echocardiogram conducted on January 5, 2023. (a) A mobile mass at the mouth of the prosthetic pulmonary valve (PPV1) from the right ventricular perspective, indicative of vegetation (arrows). (b) Substantial obstruction (peak gradient of 65 mmHg). The flow navigates around the mass, resulting in severe pulmonary regurgitation (arrows).

The patient was then admitted under pediatric cardiology on December 30, 2022, and received ceftriaxone until January 5, 2023, which was then escalated to meropenem and vancomycin until January 12, 2023, due to persistent fever. A transthoracic echocardiogram done on January 1, 2023, showed an increase in pressure gradient across the right ventricular outflow tract (RVOT) chamber from 35 mmHg in September 2022 to 65 mmHg on January 1, 2023. Therefore, the differential diagnosis of infective endocarditis was considered based on the elevated gradients. There was also a noticeable increase in the severity and gradient of the tricuspid regurgitation. Following this finding, the cardiac surgery team was consulted regarding the potential need for intracardiac echocardiography. They recommended performing a transesophageal echocardiogram (TEE) instead. Subsequent TEE on January 5, 2023, identified a mobile mass at the mouth of the prosthetic pulmonary valve, indicating vegetation with significant obstruction (see Figure 2).

*Coxiella *serology on January 12, 2023, confirmed Q fever infective endocarditis. The IgG and IgM phase two antibodies against* C. burnetti* were substantially high. Therefore, following the Centers for Disease Control and Prevention (CDC) recommendations, vancomycin and meropenem were discontinued, and doxycycline and hydroxychloroquine were commenced on the same day (January 13, 2023) [2]. The patient’s fever subsided 12 days after starting the doxycycline regimen. The patient’s case was discussed in the January 16, 2023, cardio-surgical meeting. The set plan was to continue re-evaluating the RVOT gradient for one week by TTE, and if the gradient continued to drop, subcutaneous enoxaparin would be started for the next six to eight weeks. Following that, the patient can be discharged six weeks from the start of antibiotics and scheduled for cardiac catheterization for RVOT percutaneous pulmonary valve implantation, depending on the results of a series of inflammatory markers and echocardiograms.

**Figure 3:**
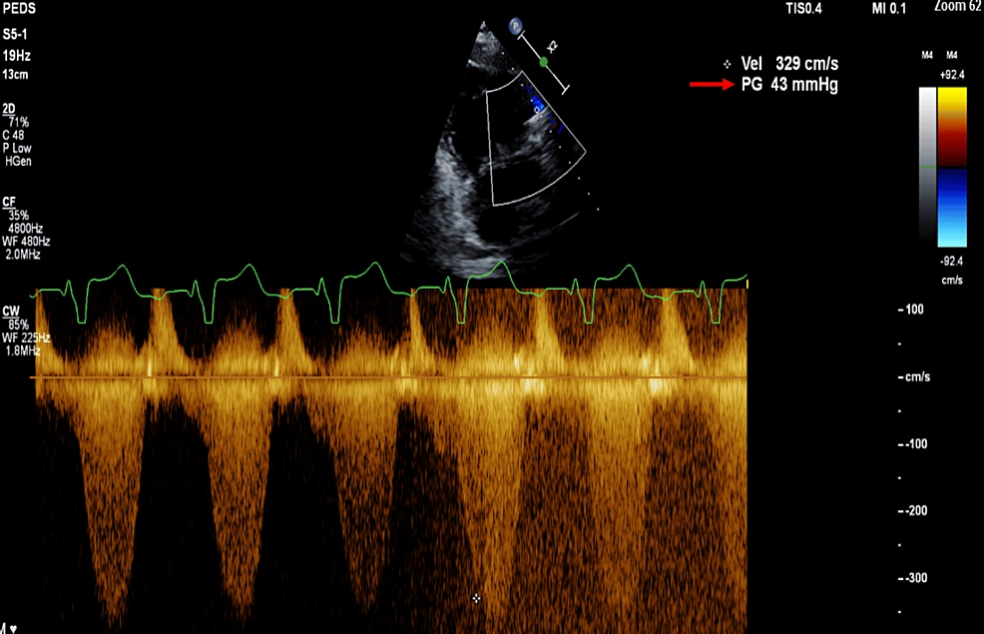
Transthoracic echocardiogram performed on January 24, 2023, indicating a reduction in the pressure gradient across the right ventricular outflow tract with an improved value of 43 mmHg.

The patient was discharged on January 26, 2023, following a 27-day hospital stay, demonstrating stability and significant improvement. TTE done on January 24, 2023, showed an improvement in the pressure gradient across the RVOT to a value of 43 mmHg (see Figure 3) compared to 65 mmHg on January 5 (see Figure 2). A comprehensive follow-up plan was established for outpatient care, including assessments such as echocardiography, CBCD, erythrocyte sedimentation rate, CRP, and blood culture. Further discussions regarding surgery were planned.

During the patient’s follow-up with the infectious disease team on March 22, 2023, the patient complained of visual disturbances, which led to the discontinuation of hydroxychloroquine and the initiation of ciprofloxacin. Following the discontinuation of hydroxychloroquine, a resolution of the patient’s thrombocytopenia was documented.

During the last outpatient infectious disease clinic visit on May 16, 2023, the patient reported feeling well with no complaints. He experienced a painless rash on his bilateral feet in March 2023, lasting for four to five days and subsequently disappearing. Currently, the patient is active, ambulatory, and tolerating oral intake without issues. He denies recent fever, respiratory symptoms, cough, or other concerns. Sleep is restful without diaphoresis. He is still on ciprofloxacin 500 mg twice daily and doxycycline 100 mg twice daily, but hydroxychloroquine was suspended on March 22, 2023. Ophthalmology periodic assessments are scheduled for ongoing monitoring. His father is satisfied with the overall improvement.

## Discussion

Although diagnosing acute Q fever in children can be challenging due to its non-specific appearance, if left untreated, Q fever can lead to chronic illnesses with severe consequences [9]. The current case raises several important clinical and management considerations.

The prevalence of Q fever infective endocarditis in Saudi Arabia remains rare. A study done in 2019 showed the number of patients that have developed Q fever infective endocarditis in 10 years from 2009 to 2018 in a tertiary cardiac center. The study showed that among the 234 patients with blood culture-negative endocarditis, 19 (8.10%) had Q fever infective endocarditis [10].

The patient’s history of pulmonary atresia, ventricular septal defect, and previous cardiac interventions made him susceptible to infective endocarditis, especially considering prosthetic valves and prior repairs. Cardiac anomalies and interventions create a conducive environment for bacterial colonization, necessitating vigilant monitoring for infectious complications [11].

Most reported studies in the literature were adult cases. To our knowledge, only one pediatric case has been reported in Saudi Arabia by Alzahrani et al. (2019); they described a case of an eight-year-old boy with congenital heart defects who initially presented with palpitation, fever, myalgia, and dyspnea. Despite negative blood cultures, Q fever endocarditis was confirmed via serology test [12]. Similarly, in our case, persistent fever and absence of remarkable findings in blood, urine, and throat cultures were reported. In contrast to the case reported by Alzahrani et al., our patient presented with additional symptoms of respiratory infection and subsequent rash development post-antibiotics.

Moreover, in our case, the positive rhinovirus/enterovirus PCR, initially overshadowing the underlying Q fever, highlights the diagnostic challenge posed by concurrent infections. Echocardiography was pivotal in the diagnosis, revealing vegetation causing RVOT obstruction. The decision to escalate antibiotic therapy, guided by the evolving echocardiographic findings, demonstrates the dynamic nature of infective endocarditis and the need for adaptability in treatment strategies.

The modified Dukes criteria is one of the best tools for diagnosing infective endocarditis [13]. It is divided into major and minor criteria, including positive blood culture of typical organisms from two separate samples and endocardial involvement. The minor criteria involve temperature >38°C, immunological phenomena (Osler’s node, Roth spots), positive blood culture not meeting major criterion, embolic phenomenon, or risk factors (congenital heart condition or intravenous drug use). Using the modified Dukes criteria for the definitive diagnosis of endocarditis, two major or one major and three minor or five minor criteria need to be met [13].

Furthermore, the treatment regimen for pediatric Q fever does not exist, and there are no specific guidelines; however, Cherry and Kersh (2021) reviewed several pediatric cases with Q fever infective endocarditis who were placed on long-term doxycycline and hydroxychloroquine therapy [9]. In addition, the CDC recommends a long-term treatment in children with chronic Q fever, including fluoroquinolone (e.g., moxifloxacin or levofloxacin) with rifampin or trimethoprim/sulfamethoxazole with doxycycline [2]. Therefore, our patient was started on doxycycline and hydroxychloroquine with periodic ophthalmology assessment. Following documented intolerance to hydroxychloroquine, it was stopped, and ciprofloxacin was added to the management plan. This underlines the individualized nature of antimicrobial regimens based on clinical response. Moreover, surgical intervention in such cases should be considered.

The involvement of cardio-surgical teams in decision-making, including discussions about potential cardiac catheterization for percutaneous pulmonary valve implantation, reflects the importance of a multidisciplinary approach in managing complex cases such as Q fever infective endocarditis [14,15]. Ongoing ophthalmology assessments and scheduled follow-ups emphasize the need for long-term monitoring for potential complications and treatment adjustments [9].

## Conclusions

This case highlights the diagnostic challenges of Q fever infective endocarditis in pediatric patients with complex cardiac histories, emphasizing the need for increased clinical suspicion. The uncommon occurrence of this condition in Saudi Arabia emphasizes the importance of tailored diagnostic approaches, especially in those with predisposing cardiac anomalies. Echocardiography played a crucial role in confirming the diagnosis and guiding treatment decisions, reflecting its significance in managing infective endocarditis. The positive response to the individualized antibiotic regimen aligns with the dynamic nature of Q fever infective endocarditis, emphasizing the need for adaptable treatment strategies.
